# 

**Published:** 1860-03

**Authors:** 


### CONTENTS FOR MARCH, 1860.

### ORIGINAL COMMUNICATIONS.

Tumors in General, and Hard Fibrous Tumors. By Prof. DEVILLE .......... 129
Interruption of Heart's Action. By B. WOODWARD, M. D. .......... 145
Trismus Nascentium successfully Treated with Sulphate of Quinine. By H. A. JOHNSON, M. D. .......... 146
Notes upon Diptheria. By J. H. HOLLISTER, M. D. .......... 148
Clinical Lecture on Erysipelas. By E. ANDREWS, M. D .......... 151
Clinical Lectures on Typhoid Fever, and Diseases of the Heart. By N. S. DAVIS, M. D., .......... 155, 160
Chicago Academy of Medical Sciences .......... 164

### BOOK AND PAMPHLET NOTICES.

Elements of Medical Jurisprudence. By Prof. T. R BECK .......... 166
Criminal Abortion in America. By HORATIO R. STORER, M. D. .......... 168
Introductory Lectures and Addresses on Medical Subjects. By GEO. B. WOOD, M. D... 169

### SELECTIONS.

Aconite and Chloroform in Neuralgia .......... 170
Alcohol and Alcoholic Preparations in Surgery .......... 171
Chloroform and Cod Liver Oil in Diptheritus and Scarlatina .......... 172
Palatable Medicines .......... 174
Alcoholic Liquors in Tubercular Diseases .......... 174
Veratrum Viride in Hemorrhage from the Gums and Tongue .......... 175
Influence of Quinine and Malaria over Pregnancy .......... 175
Revolution in Anæsthetics .......... 176
Hydrophobia successfully treated with Calomel .......... 177
Milk Sickness .......... 177

### EDITORIAL.

Improvements in Medical College Instruction .......... 179
Public Annual Commencement in the Medical Department of Lind University .......... 185
Annual Meeting of Illinois State Medical Society .......... 186
Annual Commencement of Rush Medical College .......... 187
Summer Course in Medical Department Lind University .......... 188
A Question of Ethics .......... 188
Professions against Practice .......... 191

